# Dr. Sutherland on the Treatment of Cholera

**Published:** 1849-03

**Authors:** 


*Dr. Sutherland on the Treatment of Cholera.*—Persian naphtha had been used with some effect in checking vomiting, but this medicine was so rare, that he could hardly recommend it. Chloroform had been used in the state of collapse without effect; but previous to this stage, it had been found useful in reducing cramps and spasms, and certainly relieved the sufferings of the patients. It was simply applied by being dropped on a piece of flannel, which was held to the mouth of the patient. Bleeding seemed the most effectual treatment at present. In this there was, however, a curious anomaly. At Edinburgh, in 1832, a patient was bled, and recovered, but in the next thirty-nine cases, in which bleeding was used, the patients all died. It has been tried extensively lately. Out of twelve selected cases at the Edinburgh Hospital, eleven recovered who were bled; and in one gentleman's private practice, four out of five patients recovered. This was at present a promising practice, and the results were such as to encourage its trial. But cholera was certainly an extraordinary disease; treatment which was curative one week, might prove useless on the next. Large doses of opium had been administered with good effect in some cases; but, if used too late, there was the danger of a want of reaction and the patient sinking under the effects of the remedy. The transfusion of artificial serum into the blood had not been beneficial. In the management of patients in the hospital, much depended upon their distribution. There was no evidence that the disease was contagious. In one or two instances this has been asserted; but an examination had shown it to be unfounded. It was, however, certain that cholera had a tendency to spread. In a room where there was a single patient, persons might come into contact with him in any way without danger; but if a number of patients were in one room, and persons remained there for some time, they would be liable to be attacked. This, however, was not contagion. In the Edinburgh Cholera Hospital the patients were at first widely distributed, and there was no spread o [illegible] disease. Many more patients having been received, however, the beds were crowded together; and he, (Dr. S.), on entering the wards, observed a peculiar odor, and warned the attendants of their danger. More patients were, however, received, and within twenty-four hours four of the nurses were attacked, three of whom died. Since that time fewer patients had been received; and though it was some weeks since the occurrence, none of the attendants had been attacked. This showed the importance of room and good ventilation in the wards. There was often a difficulty in obtaining external heat in the dwellings of the poor, to apply to patients. This could be done by hot bricks, water, sand, or salt. In some cases the cold water cure had been used with advantage, particularly for children,—wrapping them in a sheet dipped in cold water, and then covering them with warm clothing so as to produce perspiration; but he approved more of a plan of Professor Christison's, which was to dip a blanket into water as hot as it could be handled and wrap it round the patient. This soothed them very much, and had often been attended with good results. The great thing, however, was unremitting attention on the part of the medical men. This was particularly necessary for the poor, who, unless well looked after, were apt to neglect the prescriptions given.—*Med. Times.*—*Ibid.*

